# Morphine modulation of pain processing in medial and lateral pain pathways

**DOI:** 10.1186/1744-8069-5-60

**Published:** 2009-10-13

**Authors:** Jin-Yan Wang, Jin Huang, Jing-Yu Chang, Donald J Woodward, Fei Luo

**Affiliations:** 1 Key Laboratory of Mental Health, Institute of Psychology, Chinese Academy of Science, Beijing, China; 2 Neuroscience Research Institute, Peking University, Beijing, China; 3 Neuroscience Research Institute of North Carolina, Winston-Salem, NC, USA

## Abstract

**Background:**

Despite the wide-spread use of morphine and related opioid agonists in clinic and their powerful analgesic effects, our understanding of the neural mechanisms underlying opioid analgesia at supraspinal levels is quite limited. The present study was designed to investigate the modulative effect of morphine on nociceptive processing in the medial and lateral pain pathways using a multiple single-unit recording technique. Pain evoked neuronal activities were simultaneously recorded from the primary somatosensory cortex (SI), ventral posterolateral thalamus (VPL), anterior cingulate cortex (ACC), and medial dorsal thalamus (MD) with eight-wire microelectrode arrays in awake rats.

**Results:**

The results showed that the noxious heat evoked responses of single neurons in all of the four areas were depressed after systemic injection of 5 mg/kg morphine. The depressive effects of morphine included (i) decreasing the neuronal response magnitude; (ii) reducing the fraction of responding neurons, and (iii) shortening the response duration. In addition, the capability of cortical and thalamic neural ensembles to discriminate noxious from innocuous stimuli was decreased by morphine within both pain pathways. Meanwhile, morphine suppressed the pain-evoked changes in the information flow from medial to lateral pathway and from cortex to thalamus. These effects were completely blocked by pre-treatment with the opiate receptor antagonist naloxone.

**Conclusion:**

These results suggest that morphine exerts analgesic effects through suppressing both sensory and affective dimensions of pain.

## Background

It has been proven that systemic morphine can produce marked clinical pain relief [1,2]. Considerable advances have been made in the understanding of antinociceptive mechanisms at the peripheral and spinal levels [3,4], whereas supraspinal mechanisms are less explored. The existing data proposed that many supraspinal brain regions are involved in mediating morphine analgesia. Neuroanatomical studies have identified that several distinct opioid receptors are widely distributed throughout the central nervous system with particularly high density in limbic structures, thalamic nuclei and cerebral cortex [5-7]. Positron emission tomography (PET) studies detected the presence of endogenous opioid release and the changes of opioid receptor occupancy in cortex and thalamus during the experience of acute experimental and sustained clinical pain [8,9]. Systemic administration of opiate receptor agonists morphine or fentanyl is able to attenuate the pain-evoked responses in many supraspinal areas, such as thalamus, primary and secondary somatosensory cortex [10,11]. In the present study, we attempted to evaluate the effect of morphine on pain-evoked responses of single and ensemble cortical and thalamic neurons and on the functional interactions between cortical and thalamic areas in conscious behaving rats.

Another unsettled question about morphine analgesia is which dimensions of pain experience are most affected by opioids. It is well-known that pain is a complex experience encompassing multiple dimensions and processed by parallel ascending systems [12,13]. The 'lateral pain system' including the lateral thalamic nuclei and the somatosensory cortex is considered to be involved in analyzing the location, intensity and duration of the nociceptive stimulus, while 'the medial pain system' including the medial thalamic nuclei and the anterior cingulate cortex is associated with the unpleasant character of pain perception. Early work supported that opioid receptor agonists reduced affective-motivational dimension much more than sensory-discriminative dimension of pain [14,15]. The primary somatosensory cortex (SI) is usually considered to have the lowest opioid receptor densities and the medial pain system has a high opioid receptor density [6,16]. Nonetheless, some other studies found that opiates could reduce both sensory and affective ratings of the pain experience [17]. High opiate receptor binding potential has been found in the human lateral pain system, including frontoparietal operculum and insula [18]. Thus, the roles of medial and lateral pain systems in mediating morphine analgesia remain controversial. The second aim of the present study was to investigate how systemic morphine influences the neural activity in sensory and affective pain pathways.

## Results

### Behavioural responses

**Figure 1:**
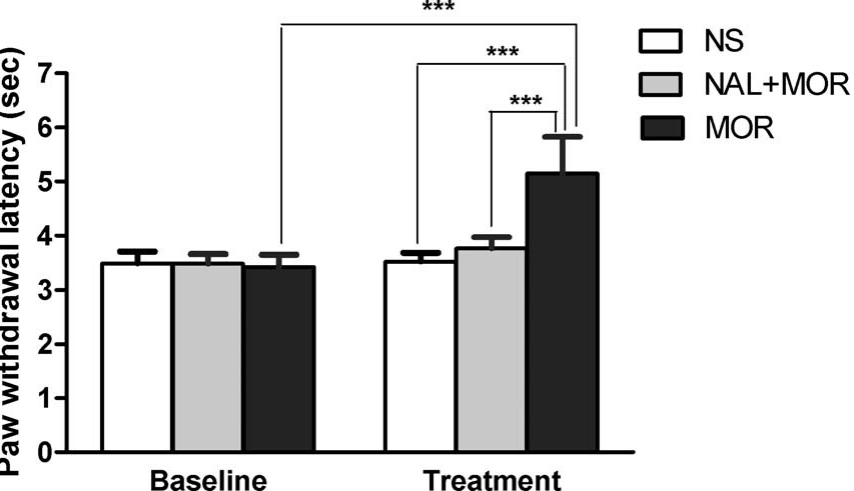
**Effect of morphine pretreatment on the thermal pain thresholds in rats**. Noxious radiant heat was used as painful stimulation, which was randomly applied to the plantar surface of the rats' hindpaws. Baseline paw withdrawal latency was measured. Morphine (5 mg/kg) or equivalent volume of NS (n = 8) was injected intraperitoneally and pain-related activities were recorded 10 min later. For NAL + MOR treatment, rats were pre-treated with 4 mg/kg naloxone 10 min prior to administration of morphine (n = 4). The ANOVA revealed that pre-treatment with morphine produced significantly longer withdrawal latency than that of any other session (*P *< 0.001). The effect of morphine was antagonized by naloxone pretreatment prior to morphine administration. NAL: naloxone; MOR: morphine; NS: normal saline.

Heat stimulation elicited apparent paw withdrawal behavior (3.49 ± 0.08 s, 3.42 ± 0.08 s, and 3.49 ± 0.09 s for the baselines of NS, morphine, and morphine plus naloxone sessions, respectively; see Fig. 1). The two-way ANOVA revealed significant session × treatment interactive effect (*F *(2, 17) = 31.87, *P *< 0.0001). Post-hoc analysis showed that pre-treatment with morphine produced significantly longer withdrawal latency (5.15 ± 0.24 s) than that of any other session (Bonferroni post tests, *P *< 0.001). The effect of morphine was prevented by naloxone pretreatment prior to morphine administration. Rats treated with saline caused no change in pain thresholds compared to the baseline level.

### General neuronal responses

**Figure 2:**
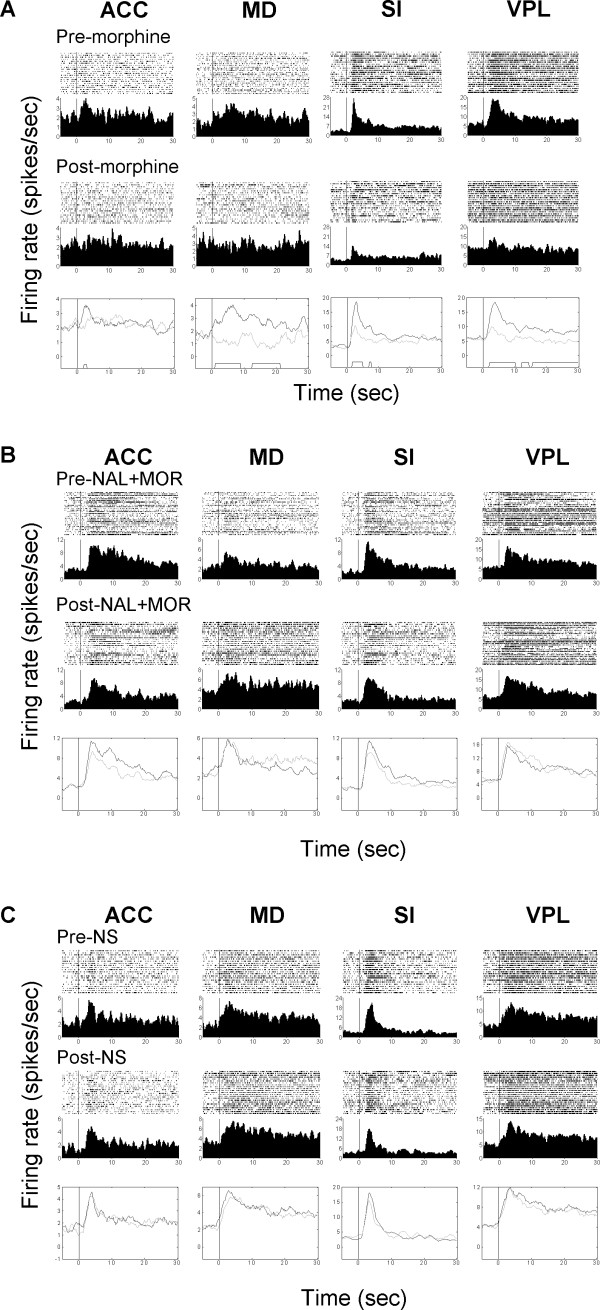
**Changes of neuronal response magnitude following morphine (A), NAL + MOR (B) or NS (C) treatment**. Raster and perievent histograms show the noxious induced responses in the four recorded areas. Figures in the top two rows in each panel illustrate typical excitatory responses before and after drug administration in each brain regions. Figures in the bottom row show the results of comparison of the pain-evoked neural activities before (black lines) and after (gray lines) morphine, NAL + MOR or NS administration. The trapezoid markers along the *x*-axis indicate the statistically significant difference between two sessions (Student's *t*-test, *P *< 0.01). Time = 0 on the axis corresponds to the time of noxious stimulation start. Note that a single dose of 5 mg/kg morphine significantly reduced the noxious heat induced activation in all the recorded areas (A), which can be reversed by naloxone pretreatment (B). No change was observed after NS injection (C). NAL: naloxone; MOR: morphine; NS: normal saline.

A total of 218 units were recorded during the morphine session (62 SI, 53 VPL, 42 ACC and 61 MD). All of these neurons were spontaneously active and the mean firing rates of SI, VPL, ACC and MD neurons were 4.15 ± 0.46, 2.53 ± 0.25, 2.74 ± 0.27, and 2.16 ± 0.25 spikes/s (mean ± S.E.), respectively. The majority of these neurons exhibited excitatory responses to noxious heat stimulation (Table 1). The pain-related neural activities within each area are shown in Fig. 2. The general response characteristics of SI, VPL, ACC and MD neurons are consistent with what we described before [19].

**Table 1:** Summary of percent of responding units according to response type

	**ACC**	**MD**	**SI**	**VPL**
Total number of neurons	42	61	62	53
Excitatory	20 (47.6%)	27 (44.3%)	48 (77.4%)	50 (94%)
Inhibitory	1 (2.4%)	2 (3.3%)	3 (4.8%)	0
Sum	21 (50%)	29 (47.5%)	51 (82.3%)	50 (94%)

### Effects of morphine on response properties of single neurons evoked by noxious stimuli

**Figure 3:**
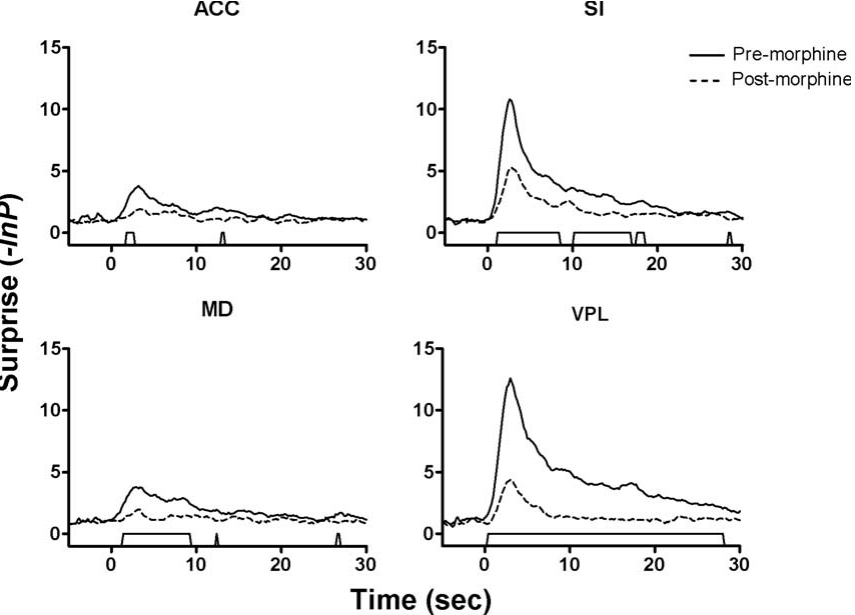
**Comparison of neuronal response magnitude before and after the administration of morphine**. Neuronal responses to noxious stimulation were evaluated using a sliding window technique, in which a 1-s time window was slid through the entire period of a trial at 1-bin step. The bin counts of each window were compared with those of a baseline window by Student's *t*-test. Only a change of firing rates from the baseline exceeds the limit of *P *< 0.01 for at least three consecutive windows were taken as a response. The '*P *values' were then converted into the information theory concept *surprise *by performing logarithmic transformation, i.e., -ln *P*. The *surprise *values were also compared using the sliding window method (1-s time window, 0.1-s step, *P *< 0.01 for three consecutive windows). Significant differences (trapezoid markers along the *x*-axis) between the response magnitude before (solid lines) and after (dashed lines) morphine administration were observed in all of the recorded areas.

The intraperitoneal injection of morphine induced a global depression of the pain-related responses in SI, VPL, ACC and MD (Figs. 2 and 3), as compared with the NS session. Fig. 2 shows an example of morphine's effect with and without pretreatment of systemic naloxone (4 mg/kg) on neuronal responses within each region. As can be seen in Fig. 2A, noxious stimulation alone evoked significant activation of thalamic and cortical neurons. Following morphine administration, the noxious heat induced responses were remarkably suppressed. These effects were always reversed by naloxone pretreatment (Fig. 2B). Injection of normal saline did not cause a change in the magnitude of pain-related response within all recorded areas (Fig. 2C). The mean pain-related responses of SI, VPL, ACC and MD neurons before and after administration of morphine are illustrated in Fig. 3. Comparison indicated that morphine significantly attenuated the neural response magnitude in all of the four areas.

**Figure 4:**
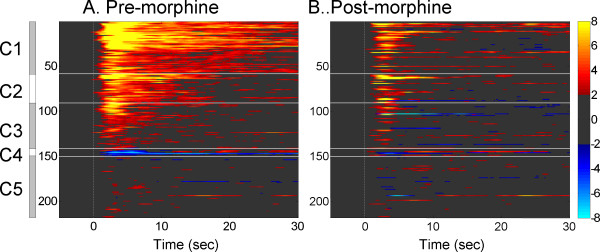
**Cluster plot depicted changes of the temporal distribution patterns of neural activity before (A) and after morphine administration (B)**. A clustering analysis was performed to classify neuronal responses depending on the similarities in patterns of excitation or inhibition around stimulation events. The firing rates were transferred into z-scores and neurons with z-scores > 2 were accepted as significantly excited and < -2 as significantly inhibited. Colorbar indicates z-scores (light yellow for highest and light blue for lowest). Each line of the image represents normalized activity of one neuron. C1-C5 represent different categories according to the response patten. The cluster analysis revealed that the cortical and thalamic areas contain units with five clusters of coding patterns in response to the noxious radiant heat (A). All of the excitatory responses were weakened by systemic injection of morphine (B).

Cluster analysis revealed that the recorded cortical and thalamic areas contain units with several different temporal coding patterns for the noxious radiant heat (Fig. 4A). Except for 7 neurons with inhibitory activities and 68 neurons without obvious responses (C4 and C5, respectively), the neurons with excitatory responses were classified as three categories according to the responding duration: long- (20-30 sec, C1), medium- (10-15 sec, C2) and short-response neurons (<10 sec, C3). Twenty-seven percent (58/218) units displayed long-duration responses, whereas 16% (35/218) had medium responses, and 23% (50/218) exhibited short responses. All of the three types of excitatory responses were shortened by systemic injection of morphine (Fig. 4B). The majority of the activation lasted no longer than 5 sec after morphine administration.

**Figure 5:**
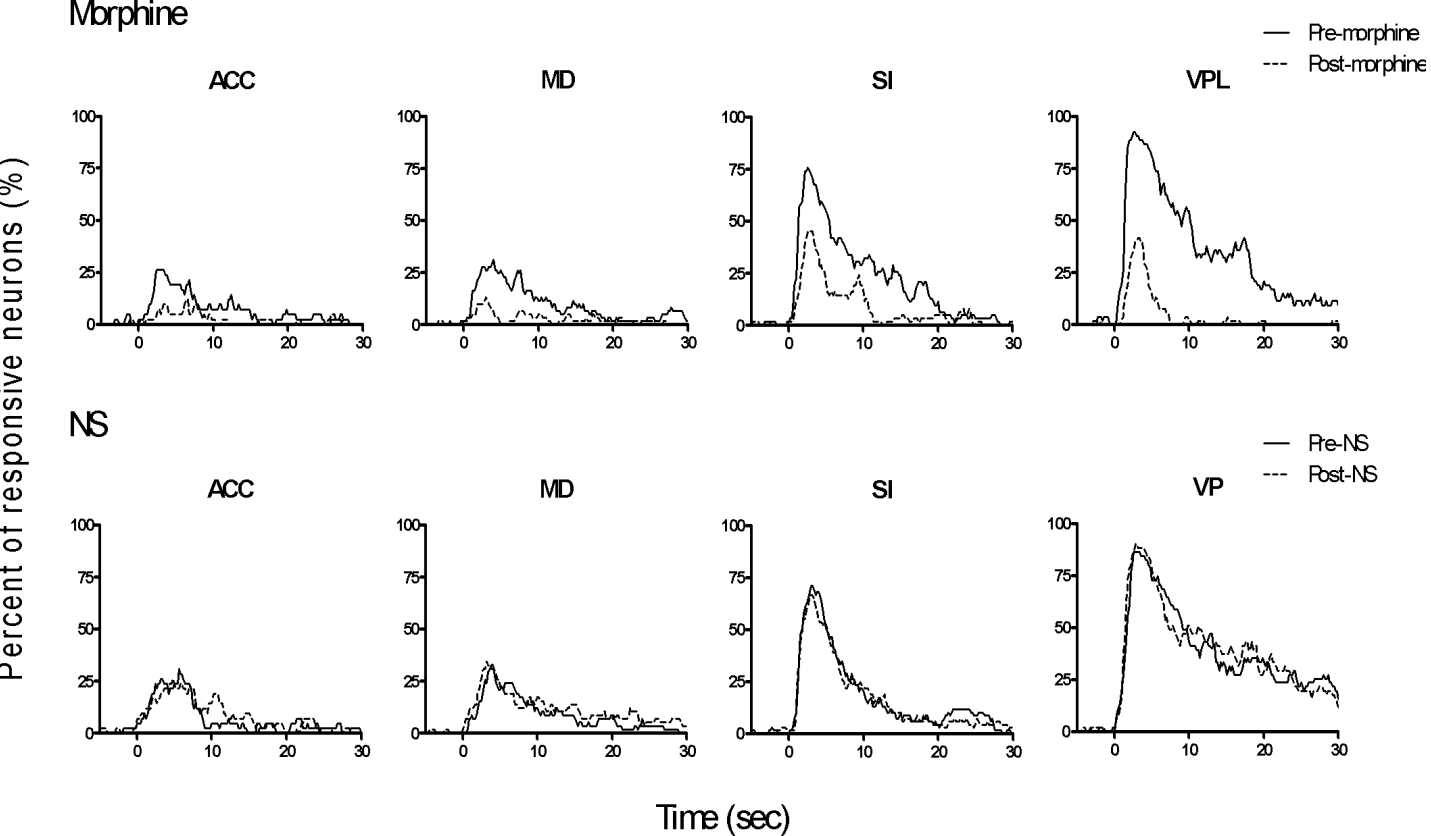
**Comparison of percentage of neurons responding to noxious stimulation before (solid lines) and after (dashed lines) morphine or NS delivery**. Chi-square tests were used to detect the percentage differences between different sessions over time. Consistent with the result of neuronal response magnitude, the number of responding neurons in all recorded areas was also decreased after injection of morphine. By contrast, NS treatment did not affect the nociceptive related responses.

In addition, morphine suppressed the activation by reducing the fraction of responding neurons. The percentage of responsive neurons in each recording area before and after injection of morphine or NS is illustrated in Table 2. There is a significant reduction in proportion of responsive neurons in the recorded cortical and thalamic areas after morphine injection. The change in percentage of responding neurons in SI, VPL, ACC and MD over time is shown in Fig. 5.

**Table 2:** Number and percentage of responsive and non-responsive neurons in each recording area before and after morphine or NS administration

			**Pre**		**Post**	
				
**Treatment**	**Brain areas**	**n**	**Responsive**	**Non-responsive**	**Responsive**	**Non-responsive**
Morphine	SI	62	51 (82%)	11	41 (66%)*	21
	VPL	53	50 (94%)	3	30 (57%)***	23
	ACC	42	21 (50%)	21	10 (24%)*	32
	MD	61	31 (51%)	30	11 (18%)***	50
	
NS	SI	69	57 (83%)	12	55 (80%)	14
	VPL	51	44 (86%)	7	47 (92%)	4
	ACC	42	22 (52%)	20	19 (45%)	23
	MD	58	28 (48%)	30	26 (45%)	32

* *P *< 0.05, *** *P *< 0.001, Chi-square test, compared with pre-treatment.

### Effects of morphine on response properties of neural ensembles evoked by noxious stimuli

#### Effects of morphine on discriminant performance of neural ensembles

**Figure 6:**
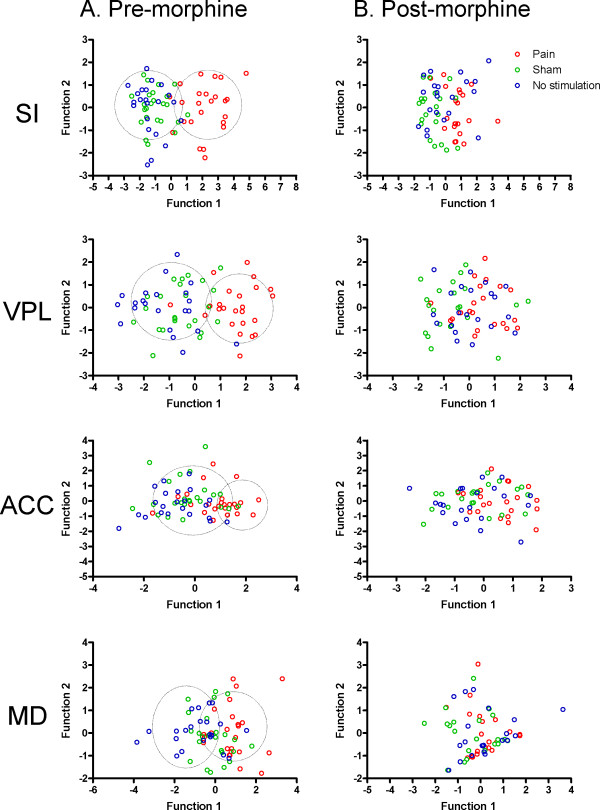
**Scatter plots depicted the capability of neural ensembles in each brain area to discriminate noxious, sham and no stimulation before (A) and after morphine administration (B)**. The linear discriminant analysis (LDA) was used to investigate whether morphine administration affected the capability of neural ensembles to discriminate different types of sensory stimulation. The noxious thermal evoked firing rates were chosen for discriminant analysis. The firing rates of multiple principle components around noxious, sham, or no-stimulation (randomly selected points where no events occurred within 30 seconds around) events were calculated and the discriminant function coefficients were estimated. As can be seen, the three categories can be well separated before the delivery of morphine (A), as indicated by the dashed line circles. In contrast, the three categories are mixed up after morphine injection (B).

**Figure 7:**
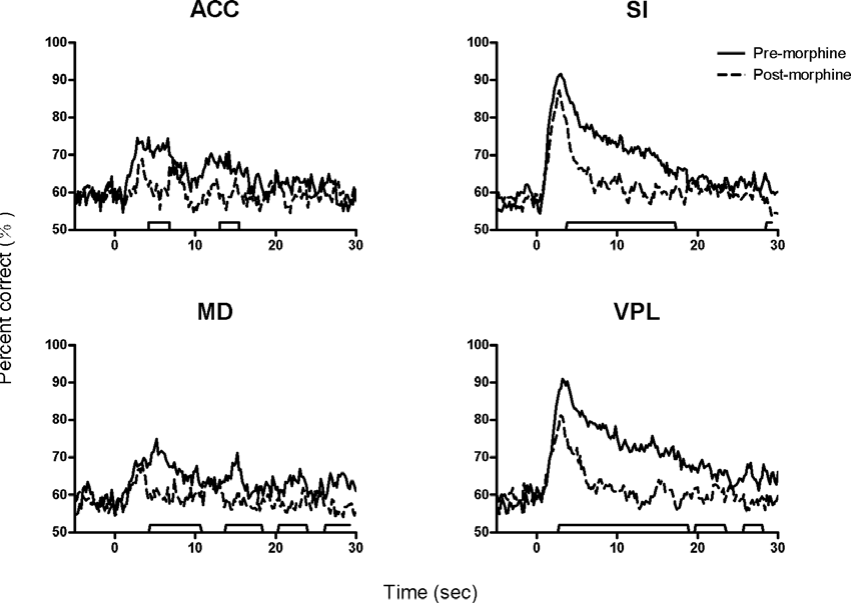
**Effects of morphine on the temporal distribution of discriminant capability of neural ensembles in ACC, MD, SI and VPL**. The correct percentage of neural ensembles to differentiate three types of sensory inputs was compared before and after morphine injection. As can be seen, the ability of neural ensembles to discriminate pain from non-pain stimuli significantly decreased after morphine administration. The markers along the *x*-axis indicate the statistically significant difference in disciminant performance before (solid lines) and after (dashed lines) injection of morphine.

The capability of neural ensembles in each brain area to discriminate noxious, sham and no stimulation before and after morphine injection is illustrated in Fig. 6. For SI and VPL, the cluster standing for noxious heat stimulation trials is well separated from the clusters standing for sham and no stimulation trials (Fig. 6A). Similarly, neurons in ACC and MD could also discriminate different sensory modalities although the discriminant capability was not as good as that of SI and VPL, demonstrated by the fact that the three clusters overlapped to a more extent than SI and VPL. This implies that neurons in the thalamocortical pathways could differentiate noxious stimulation from sham and no stimulation without pre-treatment of morphine. Interestingly, the three clusters are mixed up after morphine injection (Fig. 6B), suggesting that morphine could attenuate the ability of thalamic and cortical neurons to discriminate noxious from non-noxious stimuli. The correct discriminant percentage of neural ensembles in each recorded area was calculated and shown in Fig. 7. The ability of neurons in SI, VPL, ACC and MD to discriminate pain from non-pain stimuli after morphine injection was significantly decreased as compared to pre-injection data.

#### Change of information flow pattern by morphine

**Figure 8:**
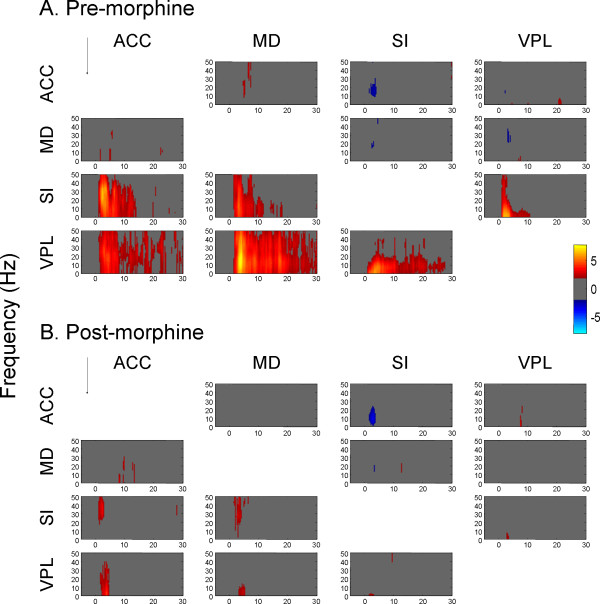
**Partial directed coherence (PDC) among different brain regions induced by noxious stimuli before (A) and after (B) injection of morphine**. The PDC values were normalized to z-scores relative to the mean and variance of baseline PDC (i.e., before noxious stimulation). The normalized PDCs exceeding 95% confident interval of the baseline were displayed in pseudo color (yellow and red for the increase and cyan and blue for the decrease). Arrows indicate the direction of information flow. (A) Before morphine injection, the amount of directed coherence from medial to lateral pathway significantly increased with a latency of 0.5 sec. (B) No significant change was observed in the PDC following morphine administration

**Figure 9:**
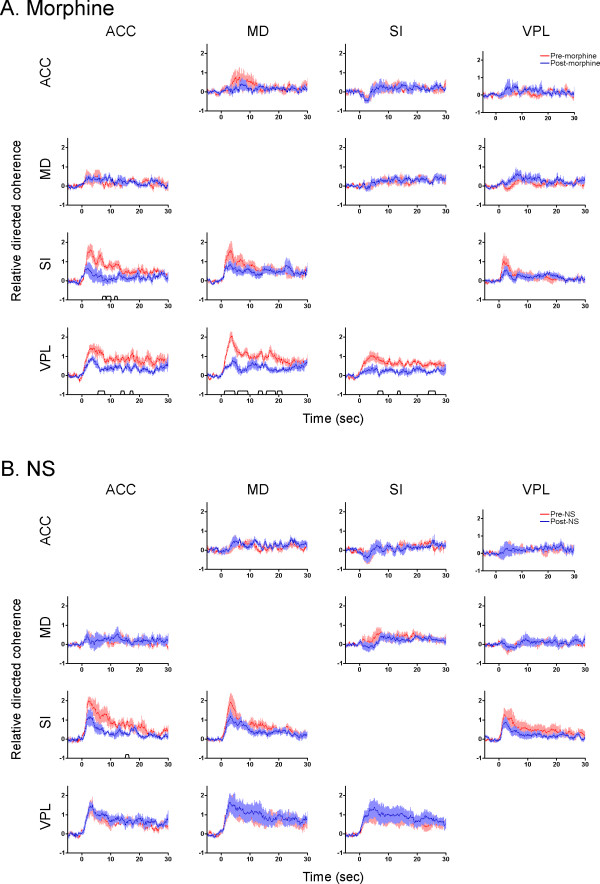
**Comparison of averaged PDC across all frequencies between pre- (red lines) and post-treatment (blue lines) of morphine (A) or NS (B)**. For each frequency band, the PDC values were normalized to z-scores relative to its mean and variance of baseline PDC. Then the PDC values of all 50 frequency bands were averaged over time. As can be seen in this figure, following morphine injection, the amount of directed coherence was significantly decreased in each direction in comparison to the pre-morphine condition. Data are presented as mean ± S.E.

The amount and direction of information flow among four recorded regions under different experimental conditions were determined using PDC analysis. The results of PDC analysis between each pair of brain areas from 8 rats are illustrated in Fig. 8. Before morphine injection, the amount of directed coherence from medial to lateral pathway significantly increased with 0.5 sec latency (Fig. 8A). The increases of the information flow lasted for at least 10 sec. By contrast, no significant change was observed in the PDC following morphine administration (Fig. 8B). The averaged PDC changes across all frequencies are shown in Fig. 9. As can be seen, the information flow in each direction was very sensitive to morphine treatment (Fig. 9A). After a single dose of morphine, only a small amount of directed coherence from medial to lateral pathway was detected. In the control session, no significant changes in the PDC values were observed after NS injection (Fig. 9B).

#### Histological localization of recording sites

Recording sites were localized by a potassium ferricyanide staining method to reveal the iron deposited at the tips of selected microwires. In the cingulate cortex, most of the iron deposits were found in the anterior areas except that in two rats the iron recording tips deflected from the target area. In the somatosensory cortex, most of the recording tips were in the hind limb region. In the thalamic nuclei, tips were mainly found in the mediodorsal and ventroposterior parts except that a VPL electrode did not reach the target depth. Electrophysiological data from electrodes that missed the targets were excluded from the analysis.

## Discussion

The present study investigated the morphine modulation of nociceptive processing in the rat medial and lateral pain pathways. The data demonstrates that: (i) at single-unit level, the increased neuronal activities in ACC, MD, SI, and VPL to noxious stimulation were significantly depressed by morphine. The depressive effects included the decrease in the neuronal response magnitude, the reduction in the fraction of responsive neurons, and the shortening of the response duration; (ii) at neural ensemble level, the cortical and thalamic capability discriminating pain from non-pain stimuli was decreased by morphine within both medial and lateral pathways. Meanwhile, morphine suppressed the pain-evoked changes in the information flow from medial to lateral pathway and from cortex to thalamus. These effects may be directly attributable to opioid receptors because they were reversed by naloxone.

Pain is an unpleasant sensory and emotional experience. Several lines of evidence suggest that the sensory and affective components of pain are mediated by separate neural pathways [13,20]. Morphine can activate opioid receptors and produce a strong analgesic effect. Numerous clinical and psychological data support that morphine profoundly reduces the emotional reaction of pain perception [14,15]. Clinical research has shown that after morphine administration, "patients frequently report that the pain is still present but that they feel more comfortable" [21]. LaGraize et al. (2006) [22] reported that both systemic administration and microinjection of morphine into the ACC produced a decrease in the aversiveness of noxious cutaneous stimulation in nerve-damaged animals, with no alteration of response to mechanical stimulation. Using the conditioned place aversion (CPA) paradigm, known to be a model for evaluating the affective component of pain in rats [23], van der Kam et al. (2008) [24] has reported that low doses of morphine are able to yield a therapeutic effect on carrageenan-induced inflammatory pain by preferentially reducing the affective component.

The present study simultaneously recorded many single units in the emotional pathways (ACC and MD thalamus) in rats under the condition of acute thermal pain, instead of employing a CPA model to specifically asses the emotion-related behavior. The results showed that the pain-related activation in ACC and MD was abrogated by morphine administration with a dose known to reduce nociceptive behavioral responses (5 mg/kg) in the rat, indicating that the medial pain system has a role in mediating morphine analgesia. ACC and MD are suggested to have particular importance in the mediation of pain emotion [20,25,26]. Patients with lesions of the ACC lost the emotional reactions to painful stimuli although pain could be further correctly localized [27]. Electrolytic lesions of MD thalamus in rats significantly decrease the avoidance behavior in the place escape/avoidance paradigm for quantifying the level of pain affect [28]. Deyama et al. (2007) [29] has provided evidence regarding the effects of morphine on the neural systems mediating the affective component of pain by showing that morphine suppresses pain-induced aversion through inhibition of glutamatergic transmission within the basolateral amygdaloid nucleus. Using both laser evoked potential and multiple single-unit recording techniques, Tsai et al. (2004) [30] demonstrated that systemic morphine significantly attenuated the long-latency but not the short-latency component of cortical responses. The long-latency component was thought to reflect C-fiber evoked response and stand for the second pain sensation (i.e., the emotional aspect of pain). Therefore, it is likely that morphine alters the affective dimension of pain through acting on the anterior cingulate cortex and medial thalamus.

ACC is a critical structure for generating emotional response, triggering behavioural withdrawal, and integrating sensory information in the processing of pain. Direct stimulation of ACC produced fear-like behaviour and induced long-term fear memory in mice [31]. Short- and long-term plasticities of ACC synaptic responses have been believed to be the cellular basis for the development of chronic pain [32-35]. NMDA receptors, particularly NR2B, in the ACC have been demonstrated to play an important role in the synaptic plasticity and the expression of fear memory [36]. Application of NR2B antagonist reduces the long-term potentiation in the ACC as well as the expression of analgesic tolerance to morphine. Chen et al (2008) found that the analgesic effect of morphine was mediated by GABAergic interneurons in addition to opioid interneurons in the ACC circuitry [37]. Although it is still unclear about the cellular and molecular mechanisms of morphine analgesia, evidence supported that morphine-induced opioid-receptor activation results in the enhancement of phosphorylation of mitogen-activated protein kinase (MAPK) in ACC, SI and locus ceruleus [38].

Another finding of the present study is that morphine significantly reduced the nociceptive related activities and discriminant ability of neural ensemble between noxious and innocuous stimuli in the lateral pain pathway (SI cortex and VPL thalamus), suggesting that opiates could attenuate the sensory aspect of pain as well. Although it has been believed that morphine has a greater effect on affective-cognitive rather than sensory-discriminative components of pain based on clinical observation, opioid receptor distribution in brain, and its effects in specific pain relay sites [39], there is psychophysical evidence that opioids can reduce both affective and sensory pain components in a dose-dependent manner [17]. Fentanyl, a μ-opioid receptor agonist, strongly attenuates noxious cold stimulation evoked responses in SI [10]. Systemic administration of morphine reduced mechanical nociception in rats with a minimal effective dose (MED) of 1 mg/kg while reduce the carrageenan-induced CPA with a MED of 0.03 mg/kg [24]. In the present study, the neuronal activities evoked by nociceptive stimuli in SI and VPL were attenuated by systemic administration of morphine, indicating an inhibition of nociceptive sensation. These results are consistent with those mentioned above as well as our previous study, in which nociceptive neural activities in SI and VPL were depressed by peripheral electrical stimulation (PES) [40]. PES exerts analgesic effect through activation of endogenous opioid system. The depression of nociceptive activities in lateral system may result from the second order effect of inhibition of medial system or activation of endogenous modulation system by morphine. This was corroborated by the observation that the information flow from medial system to lateral system significantly decreased by morphine, an indication for less influence on lateral system by medial pathway. In addition, the increased descending information flow (i.e., those from SI to VPL) elicited by noxious stimulation was attenuated by morphine. These imply that the underlying morphine analgesia mechanisms may be associated with interfering in the process of information flowing between brain nuclei. Furthermore, the present results cannot rule out the possibility that morphine has direct action on lateral system. Recent neuroimaging studies have shown that opioid receptors do not only abundantly present in the regions which are relevant to pain emotion, such as ACC and medial thalamus, but also exist in lateral pain system including somatosensory cortex in rat, dog and horse [18,41,42]. It is highly possible that morphine binds to these receptors and provides antinociceptive effect. Therefore, this study supports the notion that sensory pain component may be influenced by morphine.

A limitation of this study should be addressed here. Although PDC is a powerful tool to detect the causal relationship between brain regions that were recorded from different electrodes, the interpretations of PDC are difficult. A higher partial directed coherence does not necessarily indicate a higher coupling between the processes in brain [43]. On the other hand, it is not clear how changes in the estimated causal strength between different electrodes relate to the actual changes in the synaptic weights [44]. Thus, in the future more advanced analysis tools and a combination of varied techniques are needed to assess the complicated interactions within brain network.

## Conclusion

Our results provide evidence that morphine can inhibit nociceptive related activities in both lateral and medial pain systems. This inhibition includes attenuation of neural activation and reduction of descending control. The data imply that morphine provides relief of pain by depressing not only the affective dimension but also the sensory processing of pain.

## Methods

### Animals and surgery procedures

All experiments were performed on twelve adult male Sprague-Dawley rats (300-350 g) in accordance with the Institutional Animal Care and Use Committee of Peking University. Animals were housed individually under a reversed light/dark cycle (lights off from 7:00 A.M. to 7:00 P.M.) for 7 days before surgery. Food and water were available *ad libitum*.

Prior to chronic implants of microelectrode array, initial anesthesia was administered by an intraperitoneal injection of ketamine anesthesia (100 mg/kg, i.p.). Supplementary doses (one-third of the original) of ketamine were given as needed to maintain proper anesthetic depth during surgery. Rats were mounted on a stereotaxic apparatus (David Kopf Instruments, Tujunga, CA) and four arrays of eight stainless steel Teflon-insulated microwires (50-μm diameter, Biographics, Winston Salem, NC) were slowly lowered into primary somatosensory cortex (SI, AP = -1.0 mm, ML = 2.0 mm, and DV = 2.0 mm down; measurements relative to bregma); anterior cingulate cortex (ACC, AP = 3.2 mm, ML = 0.8 mm, and DV = 2.8 mm down); medial dorsal thalamus (MD, AP = -2.3 mm, ML = 0.8 mm, and DV = 5.5 mm down); and ventral posterolateral thalamus (VPL, AP = -3.0 mm, ML = 3.0 mm, and DV = 6.0 mm down) according to the atlas of Paxinos and Watson. Six stainless steel screws were screwed into the skull to serve as anchors for cementing the microwires in place after implantation. Animals received penicillin (16,000 U, i.m.) before surgery to prevent infection. Animals were allowed to recover for 1 week before recording sessions commenced.

### Electrophysiological recording

To record the neuronal activities, rats were placed in a plastic chamber (44 × 44 × 44 cm^3^) in a quiet room kept at 22 ± 1°C and allowed unrestricted movement during the entire recording session. Neural spike recording started after the rats become familiar with the experimental environment. Extracellular signal were collected by the chronically implanted microwire assemblies which are connected to a preamplifier via a head stage plug and two light-weight cables. The outputs of the preamplifier were filtered (0.5 and 5 kHz, 6 dB cut-off) and sent to a multichannel spike-sorting device (Biographics, Inc) for on-line signal processing. Individual waveforms were discriminated by setting multiple time-voltage windows using a PC-based software Magnet (Biographics, Inc.). The time stamps of these waveforms were then stored on a personal computer for off-line analyses. Graphical capture of waveforms, interspike interval histograms and autocorrelograms were used to validate the on-line sorting of single units. Spike train activity was analyzed with the PC-based programs *Stranger *(Biographics, Inc.) and *NeuroExplorer *(Plexon, Dallas, TX).

### Radiant heat stimulation

Noxious radiant heat from a 12.5-W projector bulb was used as painful stimulation, which was randomly applied to the plantar surface of the rats' hindpaws contralateral to the electrode implantation via a 4-mm diameter opening and through a glass floor (1 mm thick). During the experiment, a plastic frame was placed at proper position under the thin glass board, to protect it against the weight of rats. The nocifensive responses were measured by paw withdrawal elicited by the radiant heat. The focused light was manually turned off when the rat lifted the paw. Time stamps (resolution, 1 ms) of the light onset and paw lift were recorded and synchronized with the neural activities. The intensity of heat stimulus was adjusted by changing the voltage of electricity to obtain a latency within the range of 2-4 s and stimulation of identical intensity was used throughout this experiment. To avoid tissue damage, a cut-off limit of 10 s was used. To minimize sensitization and habituation, the interval between two consecutive noxious stimuli was longer than 3 min. Turning on the light without focusing on the paw was used as sham stimulation. The order of stimulus presentation was randomized. Both real and sham stimuli were delivered only when the animal was quiet and showed no voluntary motor activity. The well-isolated single unit activities were recorded simultaneously throughout the duration of all stimulation.

### Systemic administration of morphine

The effect of morphine on the pain-evoked neural responses in SI, VPL, ACC and MD were tested in 8 rats. At baseline, neuronal activities evoked by noxious heat stimulation were recorded from the abovementioned four areas. Then morphine (5 mg/kg) or equivalent volume of NS was injected intraperitoneally and pain-related activities were recorded 10 min later. The sequence of the injection of morphine or NS was random and different injections were given by a separation of ≥2 days.

To ensure specificity of the morphine effect, four animals were pre-treated with 4 mg/kg naloxone 10 min prior to administration of morphine. A repeated neuronal recording was obtained 10 min following morphine injection.

### Data analysis

Behavioral data were expressed as mean ± S.E. Statistical differences between treatments and sessions were analyzed by two-way ANOVA followed by Bonferroni post tests, with significance being defined as *P *< 0.05.

Electrophysiological data were processed offline with *NeuroExplorer *(Plexon, Inc., Dallas, TX) program for basic analysis and with *MatLab *(The MathWorks, Natick, MA) and SPSS (Chicago, IL) for advanced statistics. Bin counts per trial (0.1-s bin size) were calculated using *NeuroExplorer *program and the results were exported to Matlab in spreadsheet form. Neuronal responses to noxious stimulation were evaluated using a sliding window averaging technique, in which a 1-s time window was slid through the entire period of a trial at 1-bin step. The bin counts of each window were compared with those of a baseline window (-5~-1s) by Student's *t*-test. Only a change of firing rates from the baseline exceeds the limit of *P *< 0.01 for at least three consecutive windows were taken as a response. The '*P *values' were converted into the information theory concept *surprise *by performing logarithmic transformation, i.e., -ln *P*, to highlight the significance of the responses distributed over a time period. These values were used to plot the mean ensemble significance of neuronal responses over time. To compare neural responses between different sessions (for example, pre-morphine vs. post-morphine session), firing rates or *surprise *values were also compared using the sliding window method (1-s time window, 0.1-s step, *P *< 0.01 for three consecutive windows). The firing rates for all neurons were normalized and arranged into a spreadsheet. A clustering analysis (K-means, SPSS) was performed to classify neuronal responses depending on the similarities in patterns of excitation or inhibition around stimulation events. Chi-square tests were used to detect the percentage differences between different sessions.

Linear discriminant analysis (LDA) is a well-known method for classification and feature extraction. The aim of LDA is to find the linear combination of features which best separate several classes of objects or events. In this paper, LDA was used to investigate whether morphine affected the capability of neural ensembles to discriminate different types of sensory stimulation. The evoked firing rates were chosen for discriminant analysis. The analysis was performed using the MatLab platform and the SPSS package program. Principal component analyses (PCA) were first performed throughout the recording session for neurons within each recorded area. The firing rates of multiple principle components (PCs) around noxious, sham, or no-stimulation (randomly selected points where no events occurred within 30 seconds around) events were then exported into SPSS. The discriminant function coefficients were estimated, and the correct percentage to differentiate sensory inputs was compared before and after morphine injection.

Partial directed coherence (PDC) analysis was used to determine the amount and direction of information flow among SI, VPL, ACC and MD. The process of PDC analysis has been described in detail elsewhere [45-47]. To be brief, PDC is a frequency-domain approach of the key concept of Granger-causality to uncover the link between two neuronal groups [48]. Similar to LDA, we firstly performed principal component analyses for neurons in each brain area. Then the first principal component (PC1) of a given brain area was exported into MatLab to calculate PDC (in 1-50 Hz range). These values for each trail were then averaged. The results were normalized to Z scores relative to baseline data.

### Histology

After completion of the experiment, animals were deeply anesthetized with pentobarbital and selected tip positions of the electrodes were marked electrolytically (10-20 μA, 10-20 s, anode current). The animals were then sacrificed and perfused with 4% paraformaldehyde-5% potassium ferricyanide solution. The brains were extracted and post-fixed with the same solution used for the perfusion for 48 h. Coronal sections of 40 μm thick were cut through the SI, ACC, and thalamus. In these sections, recording sites were then determined as blue dots. Data of those sites deflecting from the target areas were out of analysis.

## List of abbreviations

ACC: anterior cingulate cortex; CPA: conditioned place aversion; MED: minimal effective dose; MD: medial dorsal thalamus; LDA: linear discriminant analysis; PCA: principal component analyses; PDC: partial directed coherence; PES: peripheral electrical stimulation; PSTHs: peri-stimulation time histograms; PWL: paw withdrawal latency; SI: the primary somatosensory cortex; VPL: ventral posterolateral thalamus

## Competing interests

The authors declare that they have no competing interests.

## Authors' contributions

JYW participated in the design of the study and helped to write the manuscript. JH carried out the experiment, and drafted the manuscript. JYC and DJW assisted with the electrophysiological recordings and data analysis. FL conceived of the study, participated in its design, assisted with the data analysis, and wrote the manuscript. All authors read and approved the final manuscript.

## References

[B1] Lorenz J, Beck H, Bromm B (1997). Cognitive performance, mood and experimental pain before and during morphine-induced analgesia in patients with chronic non-malignant pain. Pain.

[B2] Kalliomäki J, Luo XL, Yu YB, Schouenborg J (1998). Intrathecally applied morphine inhibits nociceptive C fiber input to the primary somatosensory cortex (SI) of the rat. Pain.

[B3] da Fonseca Pacheco D, Klein A, de Castro Perez A, da Fonseca Pacheco CM, de Francischi JN, Duarte ID (2008). The mu-opioid receptor agonist morphine, but not agonists at delta- or kappa-opioid receptors, induces peripheral antinociception mediated by cannabinoid receptors. Br J Pharmacol.

[B4] Reichert JA, Daughters RS, Rivard R, Simone DA (2001). Peripheral and preemptive opioid antinociception in a mouse visceral pain model. Pain.

[B5] Jones AK, Friston KJ, Qi LY, Harris M, Cunningham VJ, Jones T, Feinman C, Frackowiak RS (1991). Sites of action of morphine in the brain. Lancet.

[B6] Jones AK, Qi LY, Fujirawa T, Luthra SK, Ashburner J, Bloomfield P, Cunningham VJ, Itoh M, Fukuda H, Jones T (1991). In vivo distribution of opioid receptors in man in relation to the cortical projections of the medial and lateral pain systems measured with positron emission tomography. Neurosci Lett.

[B7] Pfeiffer A, Pasi A, Mehraein P, Herz A (1982). Opiate receptor binding sites in human brain. Brain Res.

[B8] Zubieta JK, Smith YR, Bueller JA, Xu Y, Kilbourn MR, Jewett DM, Meyer CR, Koeppe RA, Stohler CS (2001). Regional mu opioid receptor regulation of sensory and affective dimensions of pain. Science.

[B9] Petrovic P, Dietrich T, Fransson P, Andersson J, Carlsson K, Ingvar M (2005). Placebo in emotional processing--induced expectations of anxiety relief activate a generalized modulatory network. Neuron.

[B10] Casey KL, Svensson P, Morrow TJ, Raz J, Jone C, Minoshima S (2000). Selective opiate modulation of nociceptive processing in the human brain. J Neurophysiol.

[B11] Kurata J, Gyulai F, Firestone L (1999). Use of positron emission tomography to measure brain activity responses to fentanyl analgesia. Curr Rev Pain.

[B12] Albe-Fessard D, Berkley KJ, Kruger L, Ralston HJ, Willis WD (1985). Diencephalic mechanisms of pain sensation. Brain Res.

[B13] Treede RD, Kenshalo DR, Gracely RH, Jones AK (1999). The cortical representation of pain. Pain.

[B14] Rossi GC, Pasternak GW, Bodnar RJ (1994). Mu and delta opioid synergy between the periaqueductal gray and the rostro-ventral medulla. Brain Res.

[B15] Jacquet YF, Lajtha A (1973). Morphine action at central nervous system sites in rat: analgesia or hyperalgesia depending on site and dose. Science.

[B16] Vogt B, Watanabe H, Grootoonk S, Jones A (1995). Topography of diprenorphine binding in human cingulate gyrus and adjacent cortex derived from coregistered PET and MR images. Hum Brain Mapp.

[B17] Price DD, Gruen A Von der, Miller J, Rafii A, Price C (1985). A psychophysical analysis of morphine analgesia. Pain.

[B18] Baumgärtner U, Buchholz HG, Bellosevich A, Magerl W, Siessmeier T, Rolke R, Hohnemann S, Piel M, Rosch F, Wester HJ (2006). High opiate receptor binding potential in the human lateral pain system. Neuroimage.

[B19] Wang JY, Luo F, Chang JY, Woodward DJ, Han JS (2003). Parallel pain processing in freely moving rats revealed by distributed neuron recording. Brain Res.

[B20] Rainville P, Duncan GH, Price DD, Carrier B, Bushnell MC (1997). Pain affect encoded in human anterior cingulate but not somatosensory cortex. Science.

[B21] Jaffe J, Martin W, Goodman LS, Gilman A (1975). Pharmacological Basis of Therapeutics.

[B22] LaGraize SC, Borzan J, Peng YB, Fuchs PN (2006). Selective regulation of pain affect following activation of the opioid anterior cingulate cortex system. Exp Neurol.

[B23] Johansen JP, Fields HL, Manning BH (2001). The affective component of pain in rodents: direct evidence for a contribution of the anterior cingulate cortex. Proc Natl Acad Sci USA.

[B24] Kam EL van der, Vry JD, Schiene K, Tzschentke TM (2008). Differential effects of morphine on the affective and the sensory component of carrageenan-induced nociception in the rat. Pain.

[B25] Hofbauer RK, Rainville P, Duncan GH, Bushnell MC (2001). Cortical representation of the sensory dimension of pain. J Neurophysiol.

[B26] Ploghaus A, Tracey I, Gati JS, Clare S, Menon RS, Matthews PM, Rawlins JN (1999). Dissociating pain from its anticipation in the human brain. Science.

[B27] Pillay PK, Hassenbusch SJ (1992). Bilateral MRI-guided stereotactic cingulotomy for intractable pain. Stereotact Funct Neurosurg.

[B28] Wilson HD, Uhelski ML, Fuchs PN (2008). Examining the role of the medial thalamus in modulating the affective dimension of pain. Brain Res.

[B29] Deyama S, Yamamoto J, Machida T, Tanimoto S, Nakagawa T, Kaneko S, Satoh M, Minami M (2007). Inhibition of glutamatergic transmission by morphine in the basolateral amygdaloid nucleus reduces pain-induced aversion. Neurosci Res.

[B30] Tsai ML, Kuo CC, Sun WZ, Yen CT (2004). Differential morphine effects on short- and long-latency laser-evoked cortical responses in the rat. Pain.

[B31] Tang J, Ko S, Ding HK, Qiu CS, Calejesan AA, Zhuo M (2005). Pavlovian fear memory induced by activation in the anterior cingulate cortex. Mol Pain.

[B32] Shyu BC, Vogt BA (2009). Short-term synaptic plasticity in the nociceptive thalamic-anterior cingulate pathway. Mol Pain.

[B33] Zhuo M (2006). Molecular mechanisms of pain in the anterior cingulate cortex. J Neurosci Res.

[B34] Zhuo M (2007). A synaptic model for pain: long-term potentiation in the anterior cingulate cortex. Mol Cells.

[B35] Zhuo M (2008). Cortical excitation and chronic pain. Trends Neurosci.

[B36] Ko SW, Wu LJ, Shum F, Quan J, Zhuo M (2008). Cingulate NMDA NR2B receptors contribute to morphine-induced analgesic tolerance. Mol Brain.

[B37] Chen TC, Cheng YY, Sun WZ, Shyu BC (2008). Differential regulation of morphine antinociceptive effects by endogenous enkephalinergic system in the forebrain of mice. Mol Pain.

[B38] Eitan S, Bryant CD, Saliminejad N, Yang YC, Vojdani E, Keith D, Polakiewicz R, Evans CJ (2003). Brain region-specific mechanisms for acute morphine-induced mitogen-activated protein kinase modulation and distinct patterns of activation during analgesic tolerance and locomotor sensitization. J Neurosci.

[B39] Tuor UI, Malisza K, Foniok T, Papadimitropoulos R, Jarmasz M, Somorjai R, Kozlowski P (2000). Functional magnetic resonance imaging in rats subjected to intense electrical and noxious chemical stimulation of the forepaw. Pain.

[B40] Wang JY, Zhang HT, Han JS, Chang JY, Woodward DJ, Luo F (2004). Differential modulation of nociceptive neural responses in medial and lateral pain pathways by peripheral electrical stimulation: a multichannel recording study. Brain Res.

[B41] Hellyer PW, Bai L, Supon J, Quail C, Wagner AE, Mama KR, Magnusson KR (2003). Comparison of opioid and alpha-2 adrenergic receptor binding in horse and dog brain using radioligand autoradiography. Vet Anaesth Analg.

[B42] Ding YQ, Kaneko T, Nomura S, Mizuno N (1996). Immunohistochemical localization of mu-opioid receptors in the central nervous system of the rat. J Comp Neurol.

[B43] Schelter B, Timmer J, Eichler M (2009). Assessing the strength of directed influences among neural signals using renormalized partial directed coherence. J Neurosci Methods.

[B44] Cadotte AJ, DeMarse TB, He P, Ding M (2008). Causal measures of structure and plasticity in simulated and living neural networks. PLoS ONE.

[B45] Baccala LA, Sameshima K (2001). Partial directed coherence: a new concept in neural structure determination. Biol Cybern.

[B46] Sameshima K, Baccalá L (1999). Using partial directed coherence to describe neuronal ensemble interactions. J Neurosci Meth.

[B47] Fanselow EE, Sameshima K, Baccala LA, Nicolelis MA (2001). Thalamic bursting in rats during different awake behavioral states. Proc Natl Acad Sci USA.

[B48] Granger C (1969). Investigating causal relations by econometric methods and cross-spectral methods. Econometrica.

